# Gravacin as an inhibitor of the auxin transport-activating protein kinase D6PK in *Arabidopsis*

**DOI:** 10.3389/fpls.2025.1563571

**Published:** 2025-03-12

**Authors:** Meng Chen, Zhisen Yang, Yakun Peng, Lianghanxiao Sun, Xin Liu, Linfeng Sun, Shutang Tan

**Affiliations:** Ministry of Education Key Laboratory for Membraneless Organelles and Cellular Dynamics, Center for Advanced Interdisciplinary Science and Biomedicine of Institute of Health and Medicine, Hefei National Laboratory for Physical Sciences at the Microscale, Division of Life Sciences and Medicine, University of Science and Technology of China, Hefei, China

**Keywords:** auxin transport, gravacin, D6PK, PIN, arabidopsis

## Abstract

The phytohormone auxin plays a central role in plant growth and development. D6PK, a member of the AGC kinase family, phosphorylates PIN-FORMED (PIN) auxin transporters, thereby regulating PIN activity and polar auxin transport. In this study, we demonstrated that gravacin, a synthetic compound, functions as an inhibitor that targets D6PK in plants. Physiological and cell biology experiments revealed that the phenotypes of gravacin-treated plants were similar to those of *d6pk d6pkl1 d6pkl2 (d0 d1 d2)* triple mutants. Furthermore, *in vitro* kinase assays confirmed that gravacin directly inhibited the kinase activity of D6PK. Thus, by combining phenotypic analysis with cell biological and biochemical experiments, this research revealed that gravacin is an inhibitor of D6PK and elucidated the underlying mechanism. Our work provides a chemical tool that can be used to further dissect the role of D6PK and related physiological processes.

## Introduction

The phytohormone auxin plays an important role in governing nearly all facets of plant growth and patterning processes, from the embryonic stage to fruit development (Blázquez et al., 2020; Friml, 2022; Yu et al., 2022; Carrillo-carrasco et al., 2023). The directional transport of auxin is crucial for ensuring explicit plant development and tropic growth. The formation and maintenance of local auxin minima, maxima, and gradients depend on directional intercellular auxin transport, a process that is mediated by a group of auxin influx carriers, namely AUXIN1 (AUX1)/LIKE AUX1s (LAXs), and PIN-FORMED (PIN) auxin efflux carriers (Bennett et al., 1996; Vanneste and Friml, 2009; Tan et al., 2021; Ung et al., 2023). The PIN proteins consists of 10 transmembrane helices and a lengthy cytoplasmic loop, which harbors numerous phosphosites crucial for the regulation of PIN activity, polar distribution, and trafficking (Tan et al., 2021; Ung et al., 2023). In recent years, studies have confirmed that AGC (named after the cAMP-dependent [PKA] and cGMP dependent protein kinases [PKG], and protein kinase C [PKC]) kinases, mitogen-activated protein kinases (MAP kinases), calcium/calmodulin-dependent protein kinase-related kinases (Ca^2+^/CALMODULIN-DEPENDENT PROTEIN KINASE-RELATED KINASEs), and receptor kinases are capable of phosphorylating PIN proteins (Tan et al., 2021; Cui et al., 2024; Zhang et al., 2023; Luschnig and Friml, 2024; Wang et al., 2024).

D6PK, a member of the AGC kinase family, is capable of phosphorylating PIN family proteins, thereby increasing their activity in auxin transport and playing a crucial role in the directional transport of auxin (Zourelidou et al., 2009; Barbosa et al., 2014; Zourelidou et al., 2014; Weller et al., 2017). The D6PK/D6PKL subclade of AGC kinases comprise four homologous proteins, namely, D6PK, D6PKL1, D6PKL2, and D6PKL3. Loss-of-function *D6PK/D6PKL* mutants, such as *d6pk d6pkl1 d6pkl2 (d0 d1 d2)*, *d6pk d6pkl1 d6pkl3 (d0 d1 d3)*, and *d6pk d6pkl1 d6pkl2 d6pkl3 (d0 d1 d2 d3)*, present a range of auxin-deficient phenotypes, including shoot dwarfism, reduced lateral branching, defective lateral root formation, and weakened tropism (Zourelidou et al., 2009; Barbosa et al., 2014; Zourelidou et al., 2014; Weller et al., 2017). D6PK is polarly localized at the basal side of the root cell and can directly phosphorylate the intracellular hydrophilic loops of the PIN1, PIN3, PIN4, and PIN7 proteins and activate their activities, thereby promoting polar auxin transport (Zourelidou et al., 2009). Unlike the phosphorylation mediated by PINOID (PID)/WAVY ROOT GROWTHs (WAGs), the phosphorylation cascade triggered by D6PK does not impact the polar orientation of PIN proteins but rather their ability to transport auxin across the cell membrane (Zourelidou et al., 2014; Weller et al., 2017).

Specific inhibitors play crucial roles in the study of the transport mechanism of auxin. As a classic auxin transport inhibitor, N-1-naphthylphthalamic acid (NPA) has been demonstrated to bind directly and inhibit the transport activity of PIN proteins (Abas et al., 2021; Teale et al., 2021; Lam Ung et al., 2022; Su et al., 2022; Yang et al., 2022; Xia et al., 2023). Consequently, it suppresses the efflux of auxin and interferes with plant responses mediated by auxin transport. Another classic inhibitor, 2,3,5-triiodobenzoic acid (TIBA), not only directly suppresses auxin transport mediated by PIN proteins but also disrupts the trafficking of PIN vesicles and the localization of PIN proteins at the plasma membrane (Dhonukshe et al., 2008; Zou et al., 2019; Tan et al., 2020b; Xia et al., 2023). Furthermore, naproxen suppresses auxin transport, notably PIN-mediated auxin efflux, by directly binding to PIN proteins (Tan et al., 2020b; Xia et al., 2023). A range of compounds, such as endosidin4 (ES4) (Kania et al., 2018), brefeldin A (BFA) (Geldner et al., 2001; Naramoto et al., 2014), and wortmannin (Jaillais et al., 2006; Peng et al., 2024), affect the localization of PIN proteins within the endomembrane system through indirect mechanisms (Semeradova et al., 2020; Tan et al., 2021; Peng et al., 2024). These compounds have been widely used in auxin-related studies as well as in agriculture.

Gravacin was originally isolated to inhibit the negative gravitropic growth of hypocotyls in dark-grown *Arabidopsis* seedlings (Rojas-Pierce et al., 2007). Previous studies have demonstrated that ABCB19 (also called P-glycoprotein 19 (PGP19)) is a potential molecular target for gravacin, 3-(5-[3,4-dichlorophenyl]-2-furyl)-acrylic acid (PubChem compound ID 776105, Chem-Bridge ID 5850247) (Rojas-Pierce et al., 2007; Kim et al., 2010). It was proposed that gravacin plays a role in the inhibition of gravitropism by suppressing the potential auxin transport activity of the PGP19 and PGP19-PIN complexes (Rojas-Pierce et al., 2007). However, the etiolated seedlings of the *pgp19* mutants presented an overbent phenotype under dark conditions, which contradicts the proposed model in which gravacin inhibits PGP19 function (Rojas-Pierce et al., 2007). Moreover, *pgp19-4* mutants exhibit a wild-type sensitivity to the impact of gravacin on the trafficking of the tonoplast protein marker GFP-dTIP (Rojas-Pierce et al., 2007). Recent studies revealed that the ABC transporters ABCB19 and ABCB1 preferentially transport brassinosteroids rather than auxins, as supported by both biochemical assays and structural analysis (Wei et al., 2024; Ying et al., 2024). These observations suggest that a target distinct from PGP19 might be involved in this phenotype of gravacin-treated seedlings. Here, we revealed that gravacin is an auxin transport inhibitor that targets the AGC kinase D6PK, which provides novel insights into the molecular mechanisms of this compound as well as the functions of D6PK.

## Results

### Gravacin inhibited the formation of lateral roots and the negative gravitropic response of *Arabidopsis* hypocotyls in darkness

**Figure 1:**
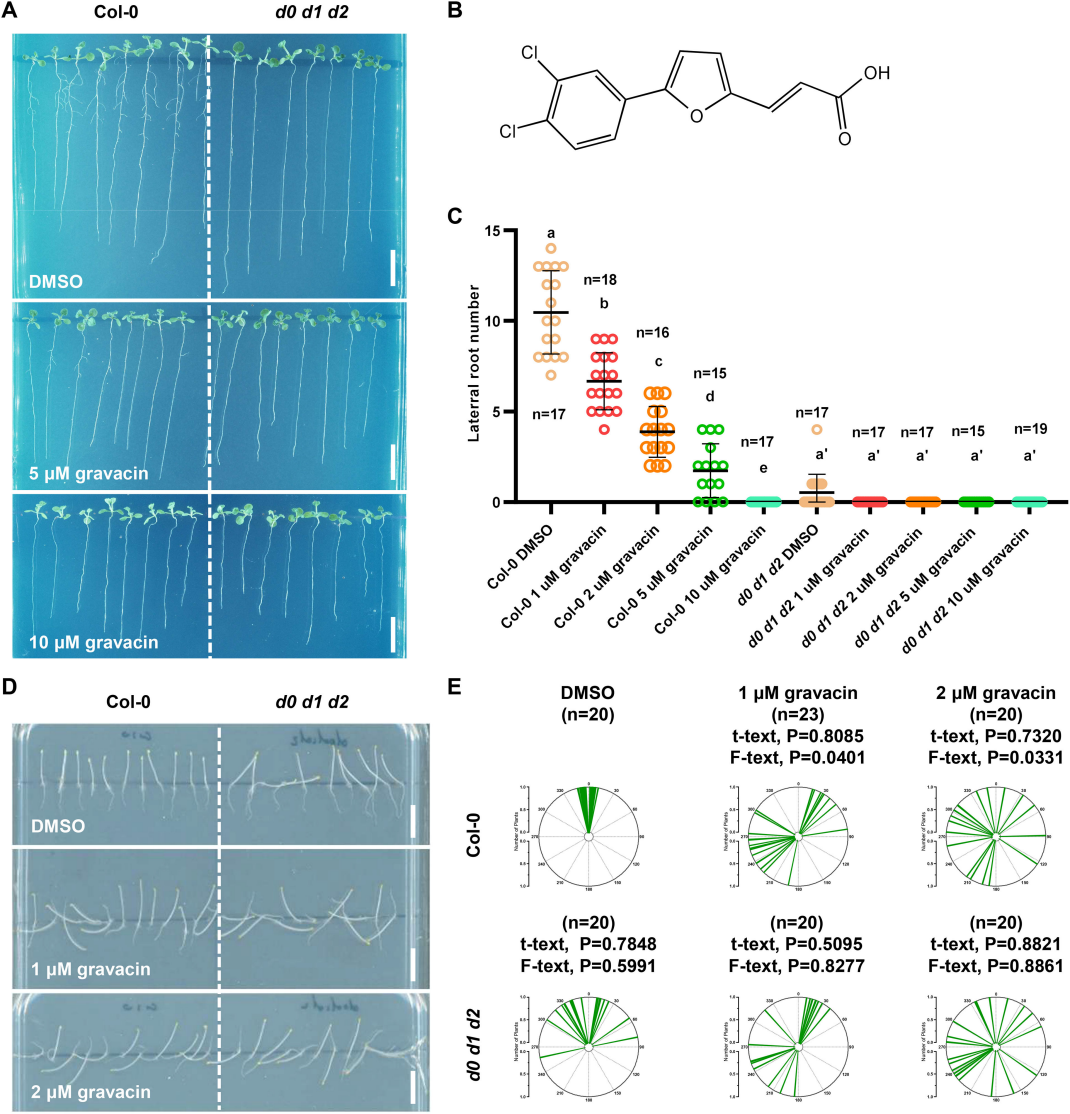
Gravacin inhibits lateral root formation and disrupts the gravitropism of *Arabidopsis* plants in the dark. **(A)**, Representative images showing the morphological changes in 11-day-old Col-0 and *d0 d1 d2* seedlings grown on MS media supplemented with gravacin. Scale bars, 1 cm. **(B)**, The structure of gravacin. **(C)**, Gravacin suppressed lateral root formation. The emerged lateral roots of 11-day-old Col-0 seedlings treated with gravacin were counted. The dots represent individual values, and the lines indicate the means ± SDs. Different letters represent significant differences; *p* < 0.05; one-way ANOVA with Tukey’s multiple comparison test. **(D)**, Gravacin interferes with hypocotyl gravitropism under dark conditions. Four-day-old etiolated Col-0 and *d0 d1 d2* seedlings were grown constantly on MS media supplemented with gravacin at the indicated concentrations. Scale bars, 1 cm. **(E)**, The angles of *Arabidopsis* hypocotyls were measured via Fiji and are shown as polar charts. Each line represents the root tip angle of an individual seedling in polar charts. Unpaired t tests indicate the difference in the mean value, and F tests indicate the difference in variance. For Col-0, gravacin treatments were compared with the DMSO control, and the *d0 d1 d2* groups were compared with Col-0 under the same gravacin treatment.

Auxin plays a major role in regulating plant gravitropism in both roots and shoots, which relies on its asymmetric distribution in response to gravi-stimulation. To delineate the molecular mechanisms underlying the functions of gravacin in plants, we investigated its physiological effects in detail. When wild-type (Col-0) *Arabidopsis* seedlings were cultivated on Murashige and Skoog (MS) plates supplemented with varying concentrations of gravacin (1, 2, 5, and 10 µM), we found that *Arabidopsis* seedlings presented shorter primary roots and a lower number of lateral roots (LRs) than did those treated with the DMSO control (**Figures 1A–C**). To study the role of gravacin in the initiation of lateral root development in detail, lateral root primordia-probed fluorescent reporters, including *pPIN1::PIN1-GFP* (Benková et al., 2003) and *pARF19::NLS-GFP* (Rademacher et al., 2012), were used for gravacin treatment. Consistently, the results revealed that the formation of lateral root primordia was significantly lower in the gravacin treatment group than in the DMSO control group (**Supplementary Figures S1A-D**).

The lateral root phenotype under gravacin treatment closely resembled that of *d0 d1 d2* triple mutants. Interestingly, *d0 d1 d2* triple mutants also exhibit defects in the negative gravitropism of hypocotyls (Zourelidou et al., 2009; Barbosa et al., 2014; Tan et al., 2020a). Consistent with these findings, when wild-type Col-0 *Arabidopsis* seedlings were grown on MS media supplemented with the DMSO solvent control in darkness, their hypocotyls grew upright. In contrast, those seedlings that grew on MS plates supplemented with gravacin presented less gravitropic hypocotyls (**Figures 1D, E**). Notably, gravacin did not affect hypocotyl length, which is consistent with the phenotype of *d0 d1 d2* (**Supplementary Figure S3B**). These findings suggest that gravacin disrupts the gravitropic response of *Arabidopsis* seedlings in darkness. To confirm these findings, 4-day-old etiolated Col-0 and *d0 d1 d2* seedlings were transferred to plates containing DMSO or gravacin, after which they were turned 90 degrees. After 24 h of gravistimulation, the etiolated Col-0 seedlings bent upward, whereas the *d0 d1 d2* seedlings bent less toward the DMSO control, and both genotypes lost gravitropism under gravacin treatment (**Supplementary Figures S2A, B**). Additionally, gravacin treatment impaired apical hook formation, similar to the defects observed in *d0 d1 d2* mutants (**Supplementary Figures S3A, C**).

These findings indicate that gravacin may interfere with the same molecular pathway as that affected in the *d0 d1 d2* mutants, which prompted us to explore whether D6PK is a potential target of gravacin.

### Gravacin treatment modulated the transcription of auxin-related genes

**Figure 2:**
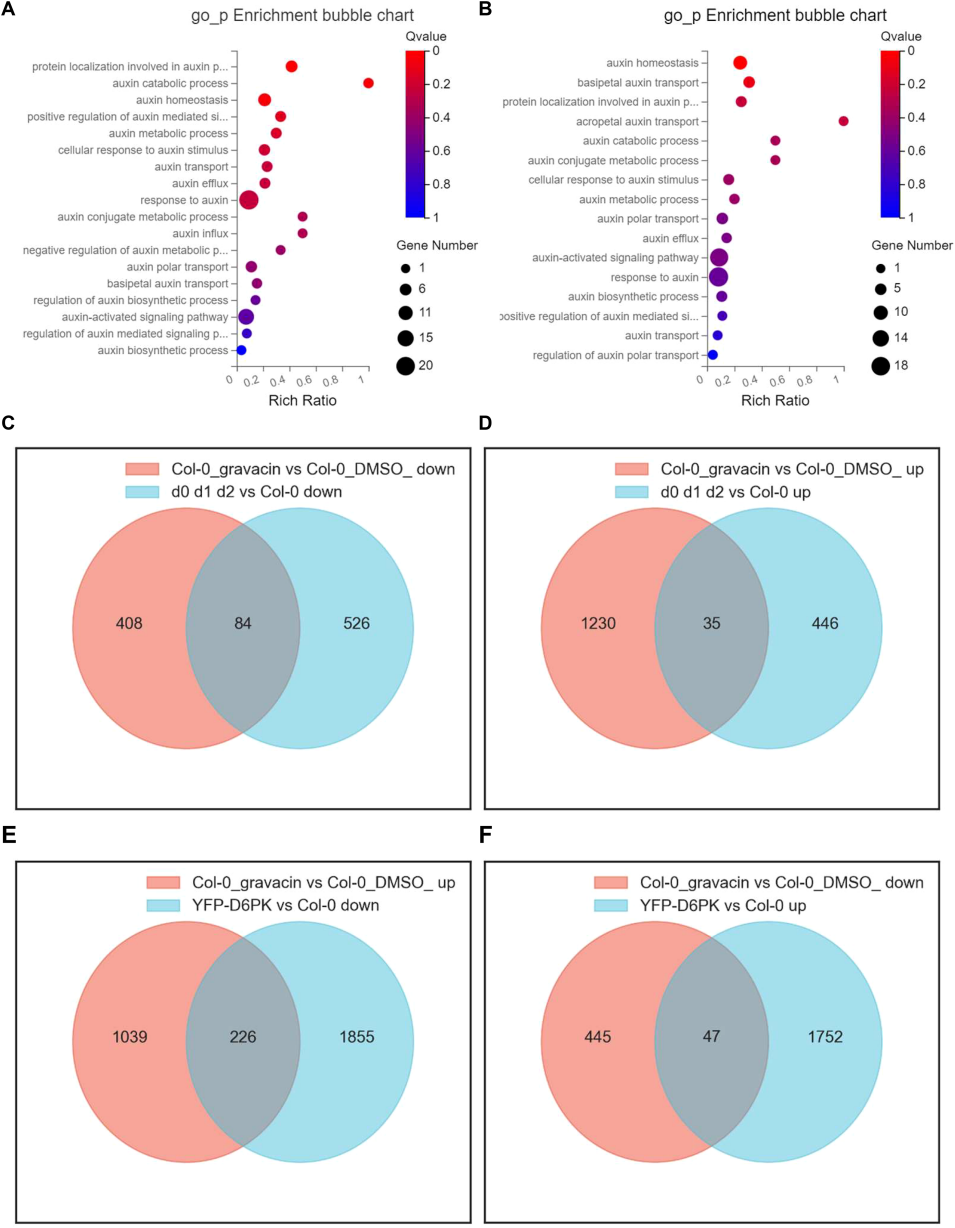
Gravacin treatment induces widespread changes in the transcriptional landscape, including the expression of auxin-related genes, in *Arabidopsis* seedlings and hypocotyls. **(A)**, GO enrichment analysis of a schematic representation of the RNA-seq data of 6-day-old Col-0 seedlings grown vertically on MS media and treated with 10 µM gravacin or DMSO (as the mock control) for 3 hours. Differentially expressed mRNAs in Col-0 seedlings treated with gravacin versus those treated with DMSO. The X-axis label represents the enrichment ratio, and the Y-axis label represents the pathway. The size and color of the bubble represent the number of DEGs enriched in the pathway and enrichment significance, respectively. **(B)**, GO enrichment analysis of a schematic representation of the RNA-seq data of 4-day-old Col-0 seedlings grown vertically in MS media in the dark and treated with 2 µM gravacin or DMSO (as the mock control). **(C)**, Venn diagram showing the overlap of downregulated DEGs in the transcriptional data of gravacin-treated etiolated Col-0 seedlings versus etiolated *d0 d1 d2* seedlings. Each colored circle represents a different dataset, and areas of overlap indicate shared DEGs. (P value < 0.05 and absolute log2 (fold change) > 0) in the translational data. **(D)**, Venn diagram showing the overlap of upregulated DEGs in the transcriptional data of gravacin-treated etiolated Col-0 seedlings versus etiolated *d0 d1 d2* seedlings. **(E)**, Venn diagram showing the overlap of upregulated DEGs in the transcriptional data of gravacin-treated etiolated Col-0 seedlings versus downregulated DEGs in etiolated *p35S::YFP-D6PK* seedlings. **(F)**, Venn diagram showing the overlap of downregulated DEGs in the transcriptional data of gravacin-treated etiolated Col-0 seedlings versus upregulated DEGs in etiolated *p35S::YFP-D6PK* seedlings.

To investigate the potential mechanisms by which gravacin exerts its physiological effects, we conducted RNA sequencing (RNA-seq) profiling of 6-day-old light-grown *Arabidopsis* Col-0 seedlings and 4-day-old dark-grown etiolated Col-0 seedlings, with each group treated with gravacin or the solvent control DMSO. Analysis of the RNA-seq data via Gene Ontology (GO) enrichment bubble plots revealed significant changes in the expression patterns of genes associated with auxin (**Figures 2A, B**), including genes related to auxin influx, auxin efflux, and the response to auxin. To evaluate the impact of gravacin on auxin signaling, we conducted RT-qPCR assays on several representative auxin-related genes. The results demonstrated that in the gravacin-treated Col-0 seedlings, there was a notable reduction in the expression of *AUX1*, *IAA6*, and *IAA19*. Conversely, the expression levels of *IAR3* (IAA-amino acid hydrolase ILR1-like 4) and *ABCB4* (ABC transporter B family member 4) were observed to be upregulated. In addition, we did not detect any change in *D6PK/D6PKL* expression under gravacin treatment in our RNA-seq data. These findings were consistent with our RNA-seq data, suggesting that gravacin modulated the auxin signaling pathway in plants (**Supplementary Figures S4A, B**).

Furthermore, Venn diagrams provided insights into the unique DEGs modulated by gravacin, as well as a distinct subset of genes that overlap with those affected in etiolated *d0 d1 d2* seedlings and in etiolated *p35S::YFP-D6PK* (Zourelidou et al., 2009) seedlings. The data revealed that 226 genes, which were upregulated in gravacin-treated etiolated Col-0 seedlings, were subsequently downregulated in etiolated *p35S::YFP-D6PK* seedlings (**Figure 2E**). Notably, the genes whose expression was downregulated by gravacin presented pronounced changes in etiolated *d0 d1 d2* seedlings (**Figures 2C, D**), suggesting that gravacin might be involved in D6PK-regulated pathways in plants.

### Gravacin interferes with the auxin response and related lateral root development and hypocotyl gravitropism in *plants*

**Figure 3:**
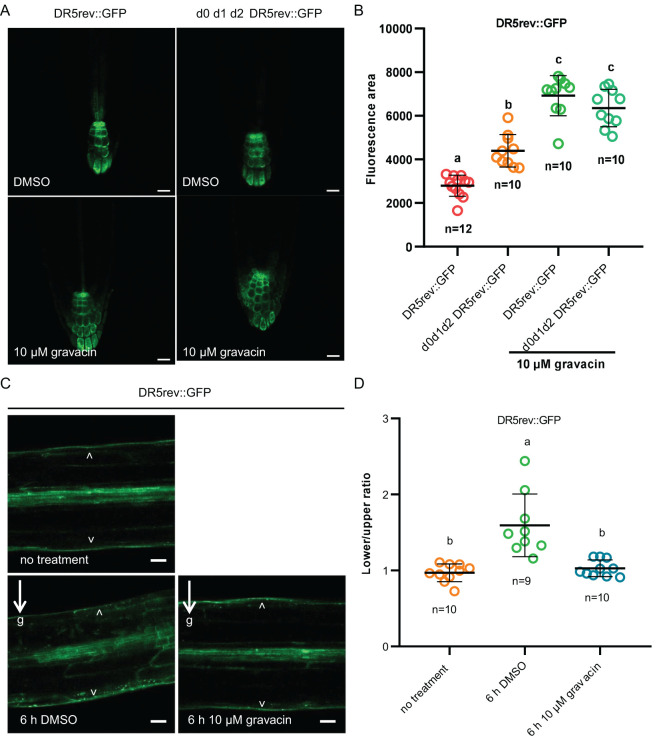
Gravacin interferes with the auxin response *in* plants. **(A)**, 5-day-old *DR5rev::GFP* seedlings or *d0 d1 d2 DR5rev::GFP* seedlings grown on MS plates containing DMSO (control) or 10 µM gravacin were imaged via confocal laser scanning microscopy, n =10-12; scale bars, 20 µm. **(B)**, The relative fluorescence intensities of *DR5rev::GFP* were measured in **(A)**. P values were calculated via one-way ANOVA with Tukey’s multiple comparison test. The dots represent individual values, and the lines indicate the means ± SDs. Different letters represent significant differences. **(C)**, 4-day-old etiolated *DR5rev::GFP* seedlings treated with DMSO (control) or 10 µM gravacin after 0 h and 6 h of gravity stimulation. The DR5 activity gradient along the gravity vector was clearly visible in *DR5rev::GFP* seedlings treated with DMSO after 6 h of gravity stimulation but not in the seedlings treated with gravacin. The arrowhead points to *DR5rev::GFP* accumulation. The arrows and “g” indicate the direction of gravity. Scale bars, 50 µm. **(D)**, Quantification of *DR5rev::GFP* fluorescence intensity in 4-day-old etiolated *DR5rev::GFP* seedlings treated with DMSO (control) or 10 µM gravacin after 0 h and 6 h of gravistimulation. The *DR5rev::GFP* fluorescence intensity was compared between the outer side of the endodermal cells at the lower and upper sides of the hypocotyls. n = 9-10, respectively. P values were calculated via one-way ANOVA with Tukey’s multiple comparison test. The dots represent individual values, and the lines indicate the means ± SDs. Different letters represent significant differences.

Previous studies have shown that D6PK controls auxin transport and distribution by phosphorylating basally localized PIN auxin transporters. Decreased activity of PIN transporters in mutants of activating kinases, such as *d6pk d6pkl1 d6pkl2* and *pdk1.1 pdk1.2*, resulted in an expansion of root tip regions with an enlarged DR5::GUS or DR5rev::GFP (an auxin responsive fluorescent reporter) region owing to the overproliferation of columellar cells (Zourelidou et al., 2009; Gao et al., 2020; Tan et al., 2020a; Xia et al., 2023). Since gravacin treatment affects genes related to auxin, we investigated whether exogenous auxin treatment could reverse the decrease in lateral root number observed in *d0 d1 d2* mutants and gravacin-treated seedlings. As expected, auxin treatment was able to rescue the lateral root phenotype in these seedlings (**Supplementary Figures S5A, B**), implying that the disruption of auxin homeostasis caused by gravacin may inhibit lateral root formation. To test this hypothesis further, we used the *DR5rev::GFP* auxin-responsive reporter (Friml et al., 2003) as an indicator of auxin distribution and signaling. Consistent with these findings, gravacin-treated roots presented broader *DR5* signal, suggesting that, compared with *d0 d1 d2*, gravacin had a similar effect on auxin distribution. (**Figures 3A, B**).

To understand the mechanism underlying the gravitropic response of gravacin-treated hypocotyls, we examined the change in auxin distribution. Analysis of the *DR5rev::GFP* reporter revealed that prior to gravity stimulation, the DR5 signal was evenly distributed on both sides of the hypocotyl. After 6 hours of gravi-stimulation, a pronounced gradient of DR5 activity along the gravity vector at the lower side was observed. However, in hypocotyls treated with gravacin, this asymmetric distribution was less pronounced (**Figures 3C, D**). These results imply that gravacin may interfere with auxin transport by regulating the activity of the D6PK protein kinase.

### Gravacin binds directly to the D6PK protein

**Figure 4:**
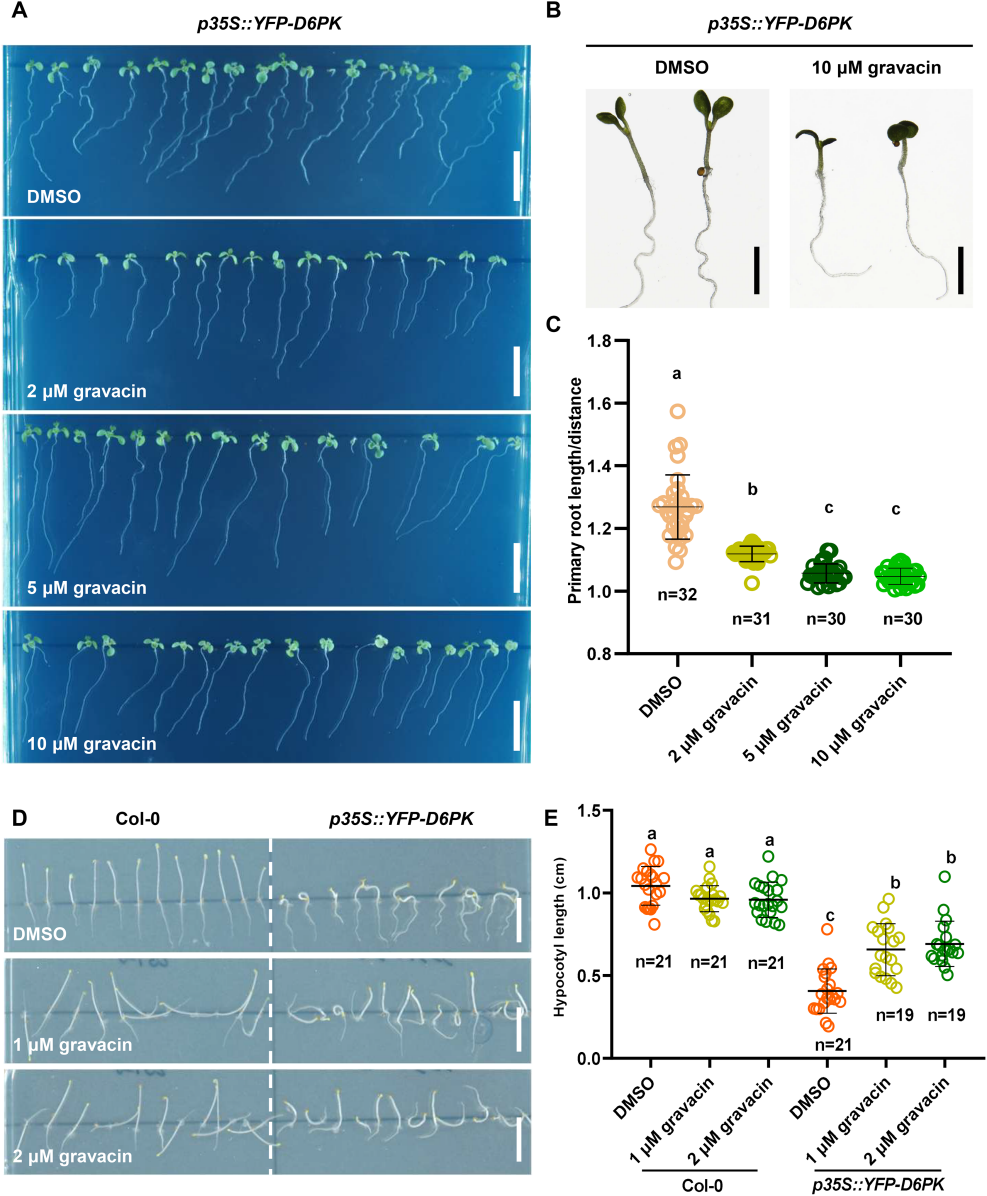
Gravacin can restore the phenotype of *p35S::YFP-D6PK*. **(A)**, 4-day-old etiolated Col-0 and *p35S::YFP-D6PK* seedlings grown on MS media supplemented with gravacin. Scale bars, 1 cm. **(B)**, The hypocotyl lengths of etiolated Col-0 seedlings and *p35S::YFP-D6PK* seedlings grown on MS media supplemented with gravacin were measured; n =19-21; P values were calculated via one-way ANOVA with Tukey’s multiple comparison test. The dots represent individual values, and the lines indicate the means ± SDs. Different letters represent significant differences. **(C)**, Seven-day-old Col-0 and *p35S::YFP-D6PK* seedlings grown on MS media supplemented with gravacin. Scale bars, 1 cm. **(D)**, Representative images of 7-day-old *p35S::YFP-D6PK* seedlings grown on plates supplemented with the indicated concentrations of gravacin were acquired with a stereomicroscope. Scale bars, 500 µm. **(E)**, Root curvature of *p35S::YFP-D6PK* could be restored by gravacin. The distance: primary root length ratio was measured to indicate the degree of bending; n=30-32; P values were calculated via one-way ANOVA with Tukey’s multiple comparison test. The dots represent individual values, and the lines indicate the means ± SDs. Different letters represent significant differences.

It was reported previously that light-grown *p35S::YFP-D6PK* seedlings have more twisted roots than wild-type seedlings do, and dark-grown seedlings have severely shortened and thickened hypocotyls. To further investigate whether gravacin potentially disrupts auxin transport by regulating the activity of D6PK, we grew Col-0 seedlings and *p35S::YFP-D6PK* seedlings on MS media supplemented with gravacin or the solvent control DMSO. Phenotypic analysis revealed that *p35S::YFP-D6PK* seedlings, when treated with gravacin, presented a reduced root wavy growth phenotype under light conditions (**Figures 4A–C**), and their shortened hypocotyls, similarly, were partially restored by gravacin in darkness (**Figures 4D, E**). These results suggest that gravacin functions through D6PK proteins.

**Figure 5:**
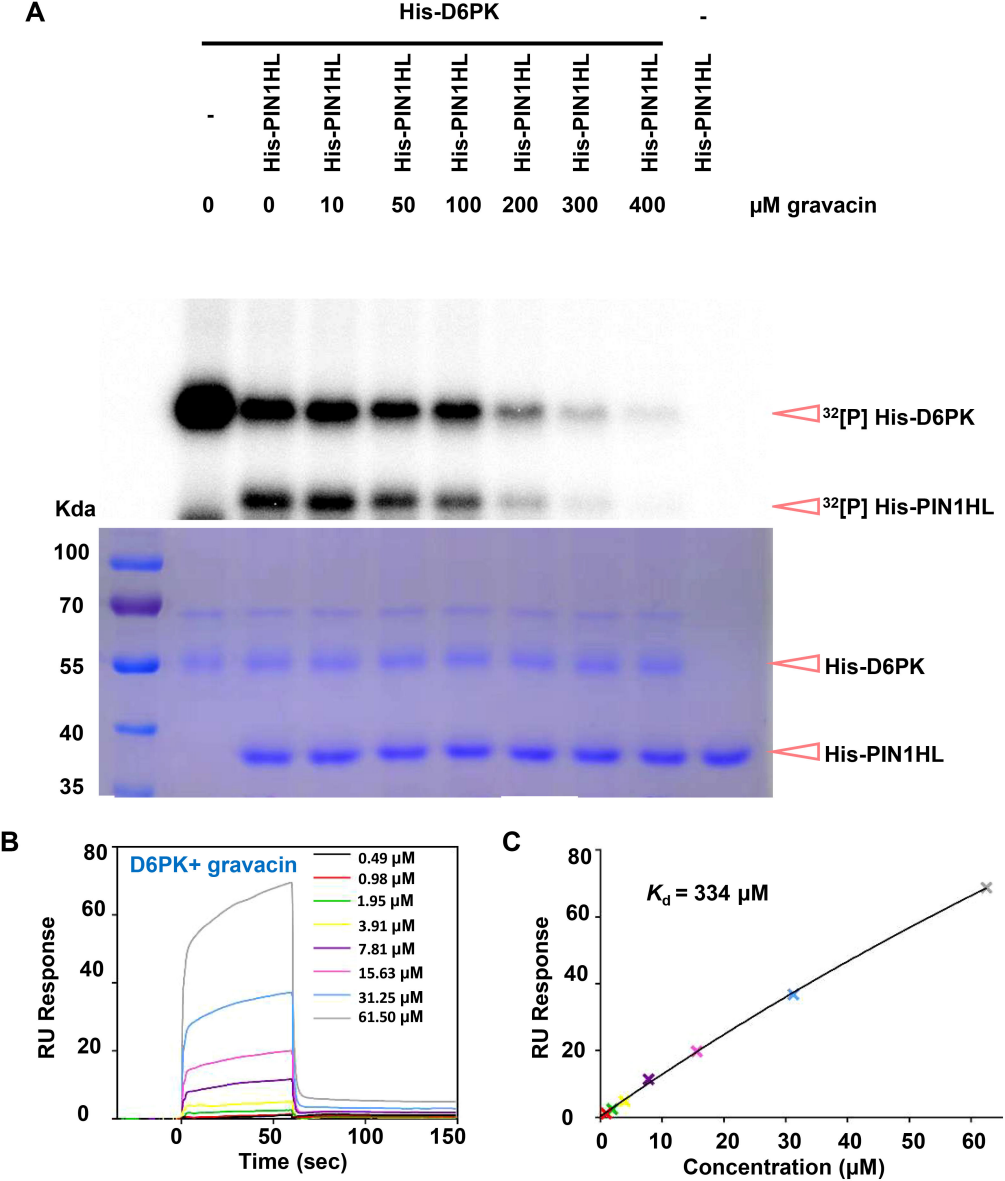
Gravacin can directly interact with D6PK and inhibit the phospholipid-D6PK-PIN cascade. **(A)**, An *in vitro* kinase assay using [^32^P] ATP revealed that His-D6PK-induced phosphorylation of His-PIN1 was partially suppressed by gravacin in a dose-dependent manner. Reactions lacking the specified components (-) were used as controls. Top, recombinant proteins were separated by 10%SDS-PAGE after incubation in protein kinase buffer containing [^32^P] ATP. Phosphorylated His-PIN1 and His-D6PK was detected by autoradiography after gel electrophoresis. Bottom, recombinant His-PIN1, His-D6PK were detected by Coomassie brilliant blue (CBB) staining. **(B)**, SPR signals for the association and dissociation of gravacin to D6PK. RU, resonance units. **(C)**, Data from **(B)** were analyzed with a steady state affinity binding model. The resulting dissociation constant *K*_d_ was determined to be 334 μM.

PINs are phosphorylated at multiple serine/threonine residues by the protein kinase D6PK. To investigate whether gravacin influences D6PK activity in phosphorylating PINs, we conducted *in vitro* kinase assays. Our results revealed that His-tagged D6PK (His-D6PK) was capable of phosphorylating His-tagged PIN1 hydrophilic loops (His-PIN1-HL) (Tan et al., 2020a). However, the addition of gravacin to the reaction system with His-D6PK led to a decrease in the phosphorylation level of PIN1-HL compared with that in the reaction with D6PK alone (**Figure 5A**). This result implies that gravacin may target D6PK protein directly. To further validate this hypothesis, we utilized surface plasmon resonance (SPR) technology to measure the binding between D6PK and gravcin. The results revealed that gravacin indeed bound to the D6PK protein, with a kinetic dissociation constant (*K*_d_) value of approximately 334 µM (**Figures 5B, C**). To gain insight into the inhibition mechanism of gravacin, we computed gravacin docking to the predicted *Arabidopsis* D6PK structure. The results indicated that the furan ring core of the gravacin was embedded within a hydrophobic cavity formed by Val124, Ala136, and Leu241, while its ortho-dichlorophenyl group located in another hydrophobic region formed by Phe188, Leu115, and Leu241, with both chlorine atoms of the ortho-dichlorophenyl group facing the solvent area. Additionally, the terminal carboxyl group of the compound might strengthen the binding stability to the target through a salt bridge interaction with the side chain amino group of Lys138, providing extra support for the overall stability of the binding conformation (**Supplementary Figures 6A, B**). In summary, the phenotypic analysis, *in vitro* kinase assays and SPR indicate that gravacin can directly binds to the D6PK, thereby inhibiting its activity and interfering with polar auxin transport.

## Discussion

This study provides compelling evidence that D6PK is a key target of gravacin. Notably, the phenotypes of gravacin-treated plants resembled those of *d0 d1 d2* mutants, which presented defects in lateral root development and compromised gravitropism. Furthermore, transcriptome analysis and cytological experiments revealed that gravacin impacts the auxin pathway, aligning with D6PK’s capacity to modulate auxin transport. Additionally, the phenotype of *p35S::YFP-D6PK* can be suppressed by the application of exogenous gravacin, suggesting that gravacin can inhibit the D6PK function *in vivo*. Finally, an *in vitro* kinase assay directly revealed that gravacin can inhibit the D6PK-PIN cascade.

Our study demonstrated that the application of a high concentration of gravacin (100 µM) effectively inhibited the D6PK-PIN1 complex, and that the binding affinity (with a *K*_d_ value of approximately 334 µM) is relatively low. In contrast, a lower concentration of gravacin (2 µM) resulted in a complete loss of the gravitropic response in etiolated wild-type seedlings. These findings suggest: 1) D6PK is likely just one of several cellular targets of gravacin, suggesting that its interference with other cellular components is essential for the disruption of gravity perception and related signaling in seedlings. 2) Gravacin may modulate protein−protein interactions of unknown proteins associated with D6PK, thereby extending its influence on cellular processes beyond auxin transport. Moreover, we cannot exclude that additional target proteins besides D6PK/D6PKLs might exist for gravacin. For example, the shortened primary root is likely mediated by additional targets other than D6PK/D6PKL1/D6PKL2, based on the remained sensitivity of the *d0 d1 d2* triple mutants to gravacin treatments.

Recent biochemical and structural studies have demonstrated that NPA is directly associated with PIN auxin transporters, thereby inhibiting their activity (Abas et al., 2021; Teale et al., 2021; Lam Ung et al., 2022; Su et al., 2022; Yang et al., 2022; Xia et al., 2023). Nevertheless, in addition to the direct interaction between NPA and PIN proteins, a variety of other protein targets have been suggested to mediate NPA function *in planta*. These include B-type ATP-binding cassette (ABC) transporters, TWISTED DWARF1 (TWD1, also known as FKBP42) and aminopeptidase 1 (APM1) (Teale and Palme, 2018). Recent studies have revealed that the ABC transporters ABCB19 and ABCB1 preferentially transport brassinosteroids rather than auxins. However, *abcb19* mutants were reported to exhibit an overbent phenotype in the dark, suggesting a potential complex role of ABCB19 in the shoot gravitropic response as well as in auxin signaling. The potential role of these non-PIN proteins as targets for NPA has been previously discussed (Teale and Palme, 2018; Kong et al., 2022). Our research identified gravacin as a direct inhibitor of D6PK. This finding offers a precise tool for dissecting the molecular mechanisms of auxin distribution, differing from the mode of action of NPA, as gravacin does not appear to interact with PIN proteins.

## Methods and materials

### Plant materials and growth conditions

The *Arabidopsis thaliana* (L.) mutants and transgenic plants were all of the Columbia-0 (Col-0) ecotype background. The transgenic marker lines *pPIN1::PIN1-GFP* (Benková et al., 2003), *p35S::YFP-D6PK* (Zourelidou et al., 2009), *pARF19::NLS-GFP* (Rademacher et al., 2012), and *DR5rev::GFP* (Friml et al., 2003) were previously reported. The mutant *d0 d1 d2* in the Col-0 background was also reported previously. A list of all of the plant lines, including mutants, reporters and crosses, is provided in **Supplementary Table S1**. A list of the primers used to genotype the T-DNA mutants is provided in **Supplementary Table S3**. For phenotyping of seedlings or pharmacological experiments, surface-sterilized seeds (using 75% ethanol) were sown on Murashige and Skoog (MS) media supplemented with 1% (w/v) sucrose and 0.8% (w/v) phytoagar (pH 5.9), stratified at 4°C for 2 d and then grown in a growth chamber at 21°C with a long-day photoperiod (16–8 h light−8 h dark).

### Pharmacological treatments

For long-term growth experiments, *Arabidopsis* seeds were sown on MS plates supplemented with the indicated chemicals. After stratification for 2 d at 4°C, the plates were moved to a growth chamber under the conditions described in the ‘Plant materials and growth conditions’ section. The phenotype of the roots was observed after an additional 7 or 11 days of growth. For lateral root induction assays, 6-day-old plants were grown on MS plates and subsequently transferred to MS plates supplemented with various concentrations of compounds. After 5 days, the number of lateral roots was counted. For PIN1::PIN1-GFP and ARF19::NLS-GFP observations, 6-day-old plants were transferred to MS plates supplemented with gravacin for 48 h. For DR5rev::GFP observation, *DR5rev::GFP* seedlings and *d0 d1 d2 DR5rev::GFP* seedlings were grown on MS plates with gravacin for 5 days. To observe the polarization of DR5rev::GFP, 5-day-old *DR5rev::GFP* seedlings grown on normal MS plates were transferred to MS plates containing gravacin and reoriented to 90°for 6 h. For the gravitropic response assay, 4-day-old etiolated seedlings were rotated 90° and subjected to gravity stimulation for a period of time before hypocotyl bending was observed. Afterwards, the samples were imaged via CLSM. A list of all of the reagents used in this study is provided in **Supplementary Table S2**.

### CLSM imaging

Images were taken via a Zeiss LSM980 CLSM instrument equipped with a GaAsP detector (Zeiss). The manufacturer’s default settings (smart mode) were used for imaging proteins tagged with GFP (excitation, 488 nm; emission, 495–545 nm) or mCherry (excitation, 561 nm; emission, 580–650 nm). All of the images were obtained at an 8-bit depth with 2× line averaging. The images were analyzed and visualized with the Fiji program (Schindelin et al., 2012; Schneider et al., 2012).

### RNA-seq analysis

For RNA-seq analysis, 6-day-old *Arabidopsis* seedlings were treated with 10 µM gravacin or DMSO for 3 h. Four-day-old etiolated Col-0 seedlings were grown constantly on MS media supplemented with 2 µM gravacin or DMSO. Each RNA sample was derived from a pool of Col-0 plants (weighing 100 mg) treated with the compounds. For subsequent analyses, three separate RNA samples for each condition were used Lists of DEGs were provided in **Supplementary Tables S4–S7**.

### RT-qPCR analysis

We utilized transcription-quantitative PCR (RT-qPCR) to investigate the transcript levels of *IAA6*, *IAA19*, *AUX1*, *IAR3* and *ABCB4* in Col-0 plants treated with 10 µM gravacin or DMSO, respectively. *ACTIN7* (AT5G09810) was used as an internal reference. In detail, total RNA was extracted from the indicated tissues using Universal Plant Total RNA Isolation Kit (Vazyme). Then, we converted 1 µg of the RNA into cDNA using the Takara kit (RR036A). The resulting cDNA of the corresponding genes and *ACTIN7* was analyzed using SYBR qPCR Master Mix (Tolobio, 22204) with a Bio-Rad CFX Connect Real-Time System. Expression values were normalized to the expression of *ACTIN7* and the data are presented as the mean ± SD. from three biological replicates.

### Protein expression and purification

The recombinant proteins His-D6PK and His-PIN1-HL (Tan et al., 2020a) were expressed in the *E. coli* strain BL21 (DE3). Protein expression was induced by 0.5 mM isopropylβ-D-1-thiogalactopyranoside (IPTG) at 16°C for 20 h. Cell lysates were derived from approximately 200 mL of *E. coli* culture and subjected to affinity purification with HisSep Ni-NTA Agarose Resin (Yeasen) according to the manufacturer’s instructions. The concentration of the purified proteins was determined via a DeNovix DS-C device.

### *In vitro* kinase assay

The procedure for *in vitro* phosphorylation assays followed a previously established protocol. Recombinant His-D6PK and His-PIN1-HL proteins were added together (5 µg of kinase and 10 µg of substrate in a 40 µL reaction system) in kinase reaction buffer (50 mM Tris-HCl, pH 7.5; 10 mM MgCl_2_; 10 mM ATP; and 1 mM DTT) in the presence of 5 µCi [^32^P]-ATP (NEG502A001MC; China Isotope & Radiation Corporation) and incubated in the presence of gravacin at 25°C for 60 minutes. The reactions were then terminated by the addition of SDS loading buffer. To terminate the reaction, SDS loading buffer was added. The samples were subsequently separated via 10% SDS−PAGE, developed overnight with a phosphor plate, and finally visualized via a Fujifilm FLA 3000 plus DAGE system.

### Surface plasmon resonance

A Biacore 8K system (Cytiva) was used to measure the binding affinity at 25°C with a flow rate of 30 µl min^−1^. Purified D6PK proteins were immobilized onto the series S CM5 sensor chips (Cytiva) by amine-coupling chemistry. Immobilized D6PK proteins were used to capture the gravacin at different concentrations. The running buffer contained HBS-EP (10 mM HEPES, pH 7.4, 150 mM NaCl, 3 mM EDTA, 0.05% surfactant P20) and 5% DMSO. Data were analyzed with the Biacore Insight Evaluation Software Version 3.0.12 using steady state affinity binding model.

### Molecular docking

The molecular docking experiment was conducted using AutoDock Vina (Version 1.2.6). Firstly, the D6PK protein structure was predicted by AlphaFold2. The compound structures were obtained by downloading from the public database PubChem and were constructed into three-dimensional structures with energy minimization using OpenBabel. The structures of the protein and compounds were further processed using AutoDockTools (version 1.5.7) to generate PDBQT format files for subsequent docking analysis by AutoDock Vina. During the docking simulation, the grid center was located at the ATP binding site of the D6PK protein to ensure effective contact between the small molecule and the target.

### Imaging and morphological analysis of *Arabidopsis* seedlings

For seedling phenotyping, photographs were captured via a Sony A6000 camera equipped with a macro lens. The primary root length was then measured with ImageJ software, and the number of lateral roots was counted manually.

### Quantification and statistics

Most experiments were repeated at least three times independently, with similar results obtained. To measure primary root length, photographs were analyzed via ImageJ (https://imagej.nih.gov/ij/download.html) (Schneider et al., 2012). The fluorescence intensity of the CLSM images was quantified via Fiji (https://fiji.sc/) (Schindelin et al., 2012). Data visualization and statistical analysis were mostly performed with GraphPad Prism 9. Polar bar graphs were generated via Origin 8.0.

## Data Availability

The sequence data from this study can be found in the Arabidopsis Genome Initiative or enBank/EMBL databases. The accession numbers are as follows: D6PK (AT5G55910), D6PKL1 (AT4G26610), D6PKL2 (AT5G47750), D6PKL3 (AT3G27580), PIN1 (AT1G73590), PIN2 (AT5G57090), PIN3 (AT1G70940), and PIN7 (AT1G23080), AUX1 (AT2G38120), IAA6 (AT1G52830), IAA19 (AT3G15540), IAR3 (AT1G51760), ABCB4 (AT2G47000). The RNA-seq data for this study are available online at the NCBI Sequence Read Archive (accession number: PRJNA1221602).
