# Supplementary material for: Gravacin as an inhibitor of the auxin transport-activating protein kinase D6PK in Arabidopsis
Source: Front Plant Sci. 2025 Mar 12;16:1563571. doi: 10.3389/fpls.2025.1563571 (PMC11938129; doi:10.3389/fpls.2025.1563571)
Supplement: Supplementary file 1 [file Presentation1.pdf]

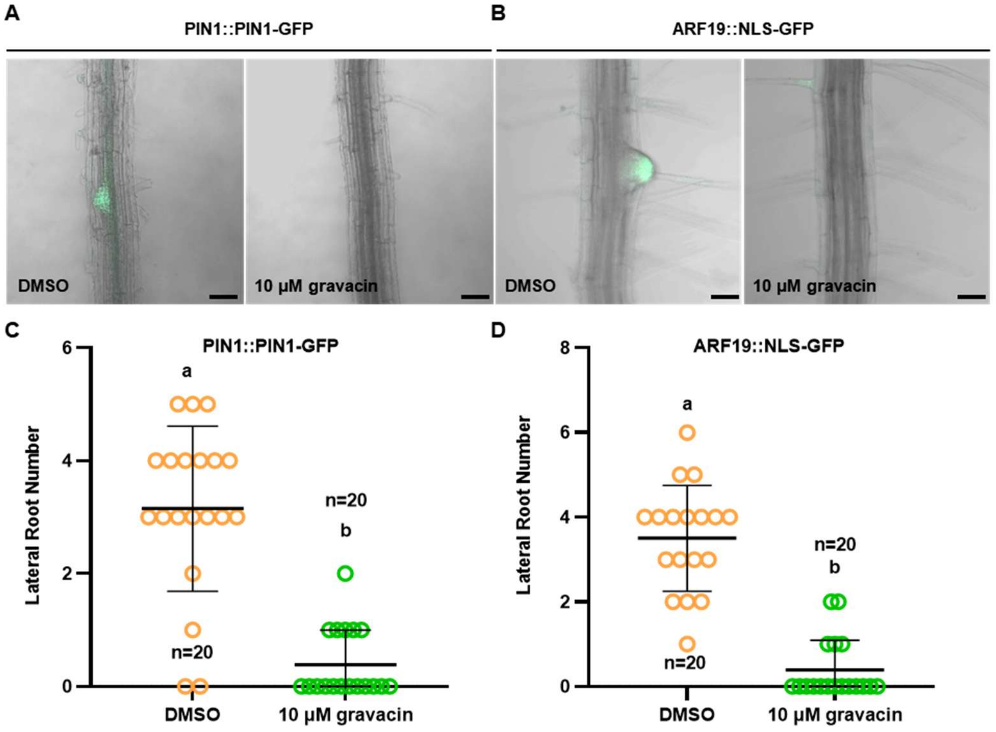

Supplementary Figure 1. Gravacin suppresses lateral root primordium formation, as demonstrated by pPIN1::PIN1-GFP and pARF19::NLS-GFP.

A, Representative images showing lateral root primordium development in the *pPIN1::PIN1-GFP* line after gravacin treatment. Six-day-old *pPIN1::PIN1-GFP* seedlings were subsequently transferred to MS media supplemented with gravacin at the indicated concentrations for an additional 48 h. Primordia were imaged via CLSM in the GFP channel (10×). Scale bars, 50 µm.

B, Representative images showing lateral root primordium development in the *pARF19::NLS-GFP* line after gravacin treatment. Six-day-old *pARF19::NLS-GFP* seedlings were subsequently transferred to MS media supplemented with gravacin at the indicated concentrations for an additional 48 h. Primordia were imaged via CLSM in the GFP channel (10×). Scale bars, 50 µm.

C, PIN1-GFP signals in the lateral root primordium were measured to indicate the number of primordia. n = 20, 20 replicates for *pPIN1::PIN1-GFP* seedlings under the indicated treatments. The dots represent individual values, and the lines indicate the means ± SDs. Different letters represent significant differences; $p < 0.05$; P values were

calculated via Welch's two-tailed t test.

D, pARF19::NLS-GFP signals in the lateral root primordium were measured to indicate the number of primordia. n = 20, 20 replicates for *pARF19::NLS-GFP* seedlings under the indicated treatments. The dots represent individual values, and the lines indicate the means ± SDs. Different letters represent significant differences; $p < 0.05$; P values were calculated via Welch's two-tailed t test.

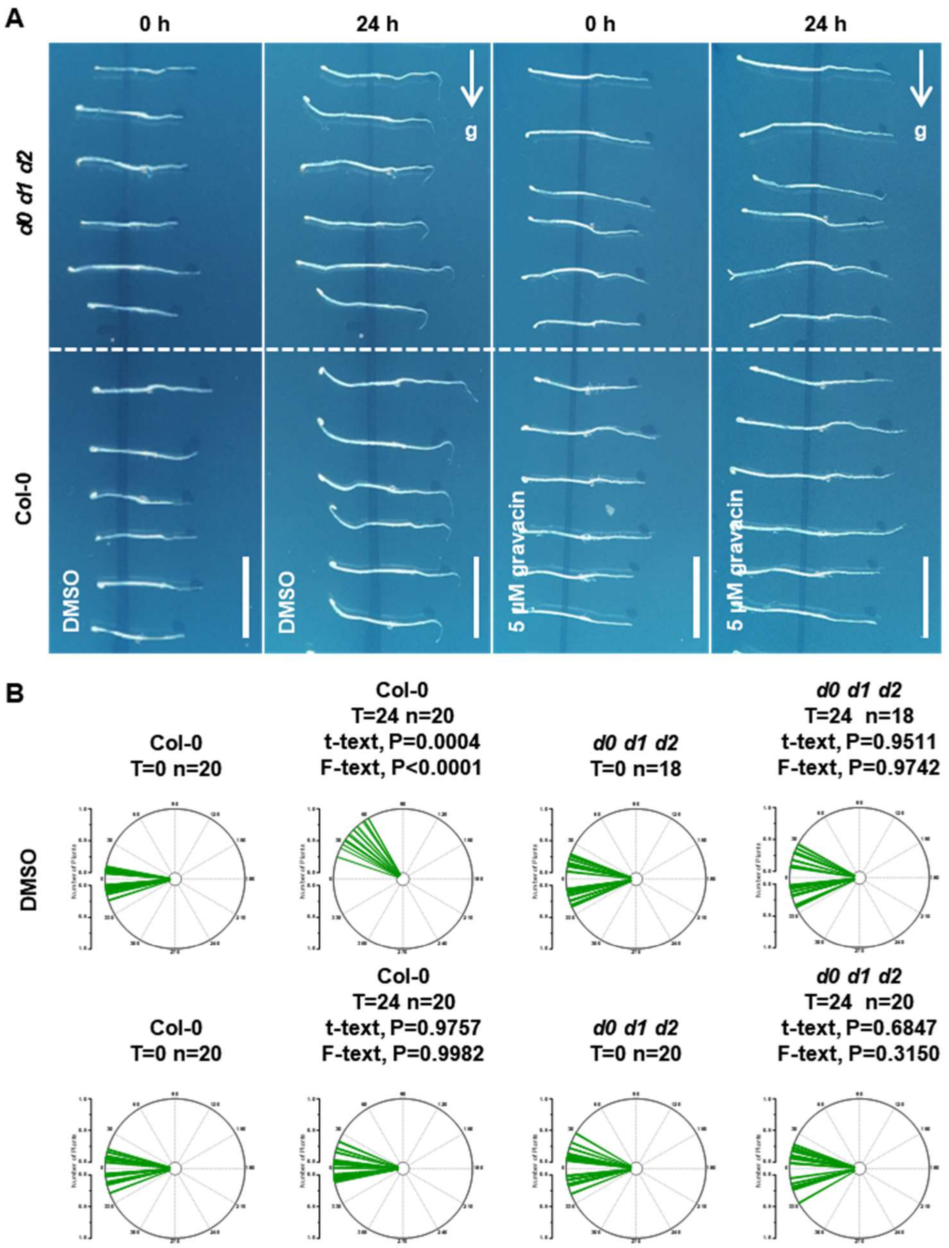

Supplementary Figure 2. Gravacin treatment impairs hypocotyl gravitropism

A, 4-day-old etiolated Col-0 and *d0 d1 d2* seedlings grown on MS media supplemented with DMSO and gravacin at the indicated concentrations were reoriented to 90°. DMSO was used as the solvent control. The arrows and "g" indicate the direction of gravity. Scale bars, 1 cm.

B, Gravacin interferes with hypocotyl gravitropism. Etiolated Col-0 and *d0 d1 d2*

seedlings (aged 4 d) were gravistimulated by 90° reorientation, and hypocotyl inclination was measured after 24 h. Each line represents the hypocotyl angle of one individual seedling in polar charts. Unpaired t tests were performed to indicate the difference in the mean value, and F tests were used to indicate the difference in variance. DMSO was used as the solvent control.

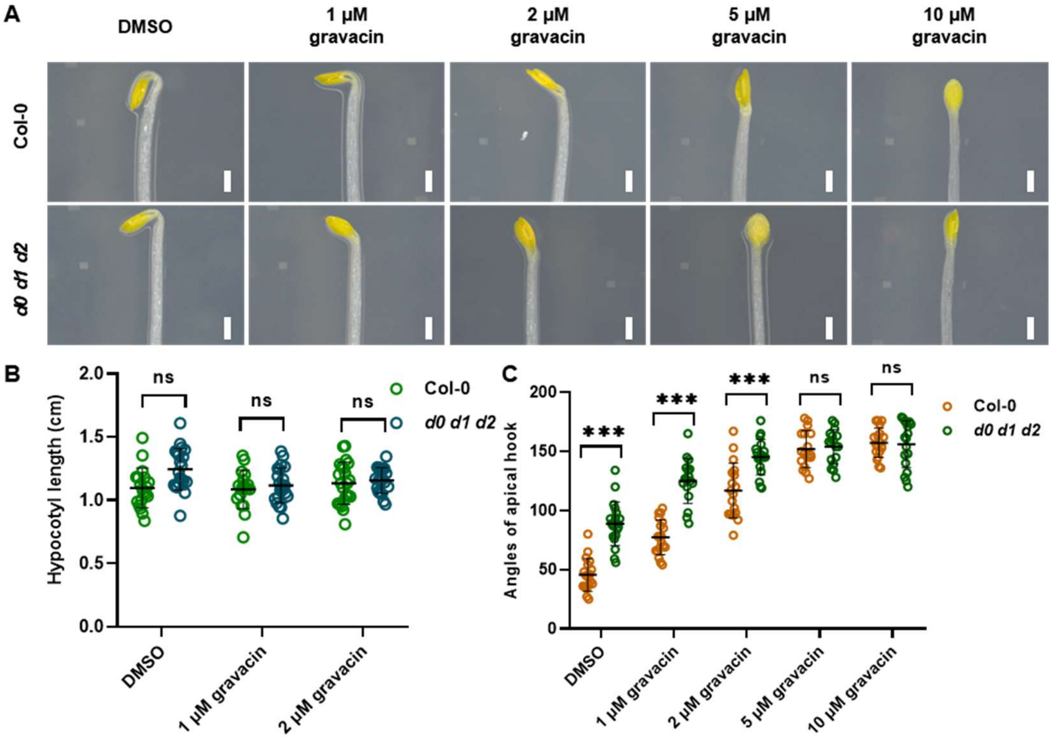

Supplementary Figure 3. Gravacin impairs the normal development of the apical hook.

A, Representative images showing the apical hooks of etiolated Col-0 seedlings and *d0 d1 d2* seedlings grown on MS media supplemented with gravacin. Scale bars, 1 cm.

B, Gravacin did not affect the length of *Arabidopsis* hypocotyls. The hypocotyl length of etiolated Col-0 seedlings and *d0 d1 d2* seedlings grown on MS media supplemented with gravacin was measured; n =18-22; P values were calculated via a two-tailed t test for the indicated pairs of Col-0 and *d0 d1 d2* at a certain concentration of gravacin. $^{**}p < 0.01$, $^{***}p < 0.001$.

C, The angle of the apical hooks of etiolated Col-0 seedlings and *d0 d1 d2* seedlings grown on MS media supplemented with gravacin was measured; n =17-20; P values were calculated via a two-tailed t test for the indicated pairs of Col-0 and *d0 d1 d2* at a certain concentration of gravacin. $^{**}p < 0.01$, $^{***}p < 0.001$.

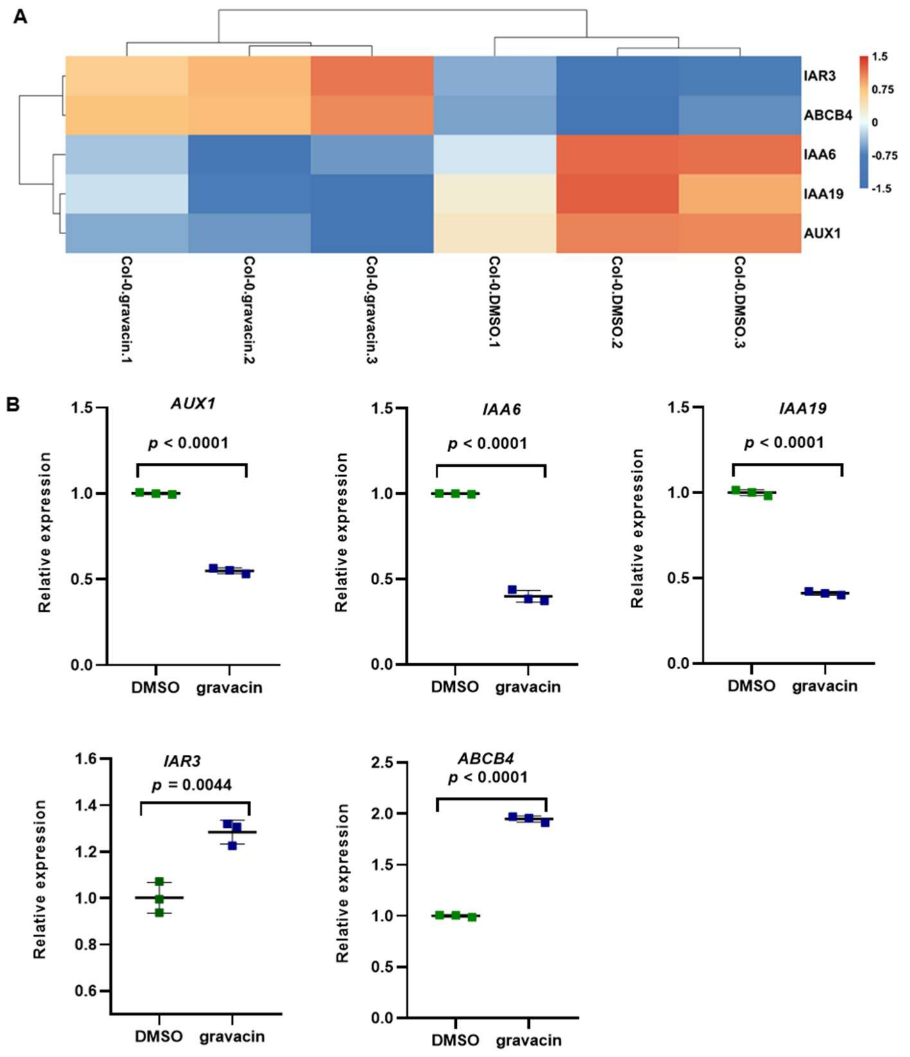

Supplementary Figure 4. gravacin treatment triggers the changes of auxin-related genes.

(A) A heatmap illustrating the expression profiles of auxin-responsive genes *AUX1*, *IAA6*, *IAA19*, *IAR3* and *ABCB4* in the Col-0 and Col-0 treated with gravacin. Six-day-old Col-0 seedlings, cultivated upright on Murashige and Skoog (MS) medium, were subjected to treatment with 10 μM gravacin or DMSO (serving as a control) for 3 h. Hierarchical clustering of log2FC values (Col-0 vs Col-0 treated with gravacin) was calculated via DESeq2 analysis.

(B) RT-qPCR analysis reveal relative expression levels of auxin-related genes *AUX1*, *IAA6*, *IAA19*, *IAR3* and *ABCB4*. Dots represent individual value and lines indicate mean ± SD. *p* values were calculated by a two-tailed t test.

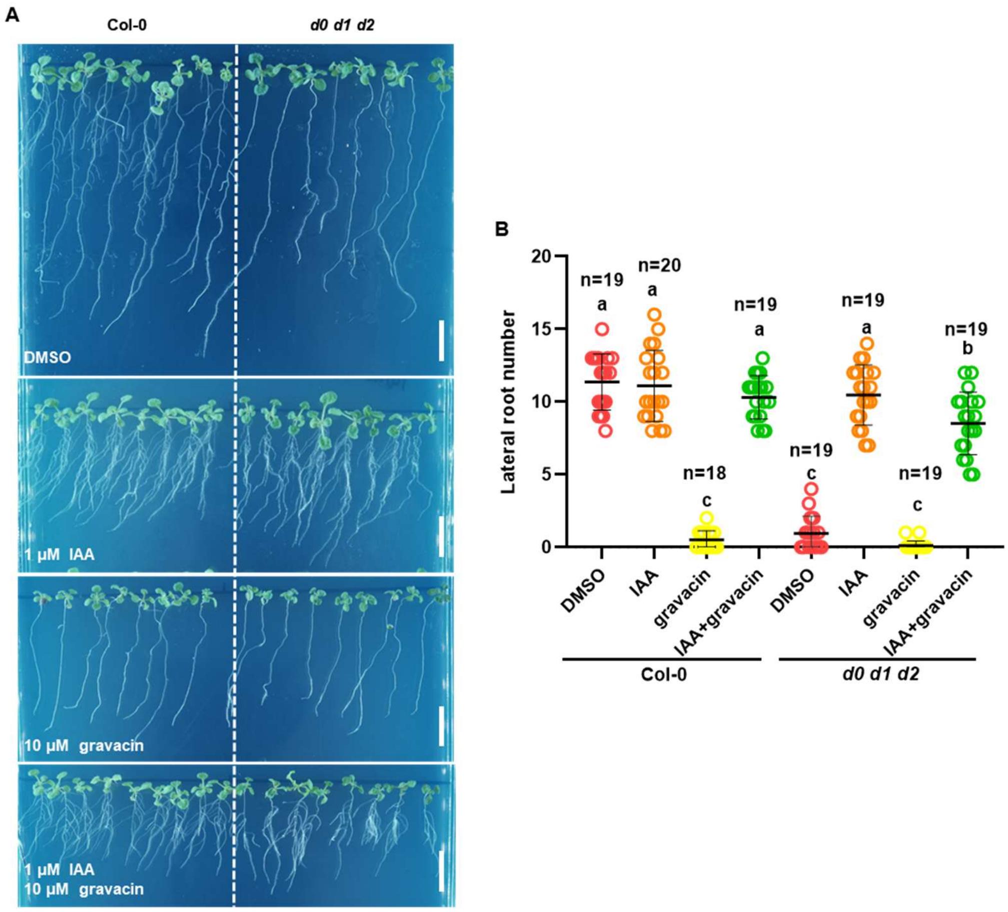

Supplementary Figure 5. The application of external auxin restores the lateral root development impaired by gravacin.

A, Representative images showing the morphological changes in 11-day-old Col-0 and *d0 d1 d2* seedlings grown on MS media supplemented with gravacin at the indicated concentrations. DMSO was used as the solvent control. Scale bars, 1 cm.

B, Application of exogenous auxin reversed the suppression of lateral root development elicited by gravacin treatment. The dots represent individual values, and the lines indicate the means ± SDs. Different letters represent significant differences; $p < 0.05$; one-way ANOVA with Tukey's multiple comparison test.

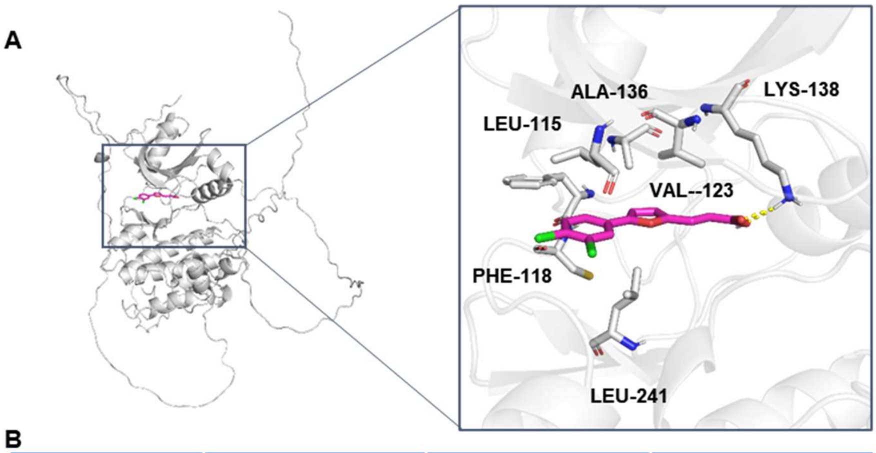

B

mode	Affinity (kcal/mol)	RMSD l.b.	RMSD u.b.
1	-7.504	0	0
2	-7.425	4.237	7.494
3	-7.386	1.385	1.996
4	-7.266	3.733	7.172
5	-6.986	4.815	8.638
6	-6.922	1.486	2.27
7	-6.838	1.846	2.118
8	-6.812	3.806	6.607
9	-6.73	2.532	3.46
10	-6.671	2.991	3.975

Supplementary Figure 6. Docking results of gravacin and D6PK.

(A) The binding pattern diagram of gravacin and D6PK. Gravacin and interacting residues are shown by sticks

(B) List of in molecular docking results for potential bindings between gravacin and D6PK.

Supplementary Table 1. List of plant lines, including mutants and maker lines, used in this study.

Arabidopsis lines	Source	Identifier
Col-0	N/A	N/A
d0 d1 d2	(Zourelidou et al., 2009)	N/A
DR5rev::GFP	(Friml et al., 2003)	N/A
d0 d1 d2 DR5rev::GFP	This study	N/A
p35S::YFP-D6PK	(Zourelidou et al., 2009)	N/A
pPIN1::PIN1-GFP	(Benková et al., 2003)	N/A
pARF19::NLS-GFP	(De Rybel et al., 2014)	N/A

Supplementary Table 2. List of reagents used in this study.

Reagent or Resource	Source	Identifier
Murashige & Skoog Basal Medium including vitamins	Duchefa Biochemie	Cat. # M0222.0050
Plant Agar	Duchefa Biochemie	P1001; CAS: 9002-18-0
Indole 3-acetic acid (IAA)	Duchefa Biochemie	I0901; CAS: 87-51-4
Dimethyl sulfoxide (DMSO)	Sigma-Aldrich	D4540; CAS: 67-68-5
3-(5-[3,4-dichlorophenyl]-2-furyl)-acrylic acid (gravacin)	ChemBridge	CAS:188438-05-3
**Bacteria**		
Escherichia coli strain BL21	Vazyme	Cat# C502-02
**Kits**		
GeneJET Plasmid Miniprep Kit	Thermo Fisher Scientific	Cat. # K0503
**Software and Algorithms**		
Arabidopsis Information Resource (TAIR)	N/A	https://www.arabidopsis.org/
image J	image J	https://imagej.nih.gov/ij/
Fiji	Fiji	https://fiji.sc/
ZEN	ZEN	https://www.zeiss.com/microscopy/en/products/software/zeiss-zen-lite.html
GraphPad Prism 8.3.0 (538)	Graphpad Software, USA	https://www.graphpad.com/scientific-software/prism/
Origin 2023	OriginLab	https://www.originlab.com/

Supplementary Table 3. List of primers used in this study.

**Primers**	**Oligonucleotide (5' to 3')**	**Purpose**
**For Genotyping T-DNA Insertional Mutants**		
**Oligo Name**	**Sequence**	**Allele**
d6pk-LP	TGAGAATCATCAACTGTGGAAAC	*d6pk-1*
d6pk-RP	TTTGGTGATGGAGTTTTGTCC	
d6pkl1-LP	TCTCTTCATTTCCATGGAAGG	*d6pkl1-1*
d6pkl1-RP	CTCCAGTTTTACTGTCGCTGC	
d6pkl2-LP	CTTCGCCTTTGATGATCTCTG	*d6pkl2-2*
d6pkl2-RP	AGTGACGAGAGTAGCTGCAGC	
d6pkl3-LP	CCATTAAACGACGAAACATCG	*d6pkl3-2*
d6pkl3-RP	TAACAAGCTTCTTCCTCGCTG	
LBb1.3	ATTTTGCCGATTTCGGAAC	SALK lines
**For RT-qPCR**		
IAA6-FP	GCGAAATATCAGTATGCGGATC	
IAA6-RP	CAGTATCTAACGCTGATGAGGT	
AUX1-FP	CCTAAGCAATTTCCTATGGCAC	
AUX1-RP	GCTCTGTATTCGACGTAGAGAA	
IAR3-FP	TTCACTTGCTTAATCCGACTCT	
IAR3-RP	TCTCCAACTCTGCTCTAACAAG	
ABCB4-FP	CAGAGATTCCGGTGCTAATACT	
ABCB4-RP	GAAAAACGCTTTGATTACGCTG	
IAA19-FP	CGCTGAGAAGGTTAATGATTCG	
IAA19-RP	TCACTTTCACATACCCTAACCC	

References

Benková, E., Michniewicz, M., Sauer, M., Teichmann, T., Seifertová, D., Jürgens, G., and Friml, J. (2003). Local, efflux-dependent auxin gradients as a common module for plant organ formation. Cell **115,** 591-602.

De Rybel, B., Adibi, M., Breda, A.S., Wendrich, J.R., Smit, M.E., Novák, O., Yamaguchi, N., Yoshida, S., Van Isterdael, G., Palovaara, J., Nijsse, B., Boekschoten, M.V., Hooiveld, G., Beeckman, T., Wagner, D., Ljung, K., Fleck, C., and Weijers, D. (2014). Plant development. Integration of growth and patterning during vascular tissue formation in *Arabidopsis*. Science **345,** 1255215.

Friml, J., Vieten, A., Sauer, M., Weijers, D., Schwarz, H., Hamann, T., Offringa, R., and Jürgens, G. (2003). Efflux-dependent auxin gradients establish the apical-basal axis of *Arabidopsis*. Nature **426,** 147-153.

Zourelidou, M., Müller, I., Willige, B.C., Nill, C., Jikumaru, Y., Li, H., and Schwechheimer, C. (2009). The polarly localized D6 PROTEIN KINASE is required for efficient auxin transport in *Arabidopsis thaliana*. Development **136,** 627-636.
